# PARP Inhibition in Prostate Cancer: Current Status, Resistance Mechanisms, and Clinical Challenges

**DOI:** 10.3390/cells15070588

**Published:** 2026-03-26

**Authors:** Takashi Matsuoka, Shusuke Akamatsu, Christopher J. Ong, Martin E. Gleave, Yuzhuo Wang

**Affiliations:** 1 Vancouver Prostate Centre, Vancouver General Hospital, Vancouver, BC V5Z 1M9, Canada; tmatsuoka@bccrc.ca (T.M.); chris.ong@ubc.ca (C.J.O.); 2 Department of Experimental Therapeutics, BC Cancer Agency, Vancouver, BC V5Z 1L3, Canada; 3 Department of Urologic Sciences, Faculty of Medicine, The University of British Columbia, Vancouver, BC V5Z 1M9, Canada; 4 Department of Urology, Graduate School of Medicine, Kyoto University, Kyoto 606-8507, Japan; 5 Department of Urology, Graduate School of Medicine, Nagoya University, Nagoya 466-8550, Japan; akamats@med.nagoya-u.ac.jp

**Keywords:** PARP inhibitors, homologous recombination repair (HRR), BRCA1/2, androgen receptor pathway inhibitors (ARPI), androgen deprivation therapy (ADT), metastatic castration-sensitive prostate cancer (mCSPC), metastatic castration-resistant prostate cancer (mCRPC), combination therapy, biomarkers

## Abstract

**Highlights:**

**What are the main findings?**

- PARP inhibitors show the most consistent benefit in advanced prostate cancer with BRCA1/2 alterations, especially BRCA2.
- Resistance is driven by mechanisms such as HRR restoration (reversions) and replication-stress tolerance and may involve dormant/quiescent, therapy-tolerant residual tumor-cell states.

**What are the implications of the main findings?**

- Better biomarkers and monitoring (functional HRR assays, ctDNA) are needed to guide patient selection and detect resistance early.
- Testing PARP inhibitor-based combinations earlier in mCSPC and targeting minimal residual disease may broaden the benefit and delay relapse.

**Abstract:**

Poly(ADP-ribose) polymerase inhibitors (PARPi) have reshaped therapy for advanced prostate cancer, yet durable benefit remains concentrated in BRCA1/2-altered tumors, especially BRCA2, and most responders eventually relapse. Here, we frame PARPi response and resistance through a unifying model in which DNA damage response (DDR) rewiring (e.g., homologous recombination repair (HRR) restoration, fork protection, checkpoint tolerance, and altered drug handling) converges with treatment-induced dormancy and quiescent therapy-tolerant residual states that sustain minimal residual disease (MRD) under androgen receptor pathway inhibition (ARPI) and PARP blockade. We synthesize clinical and translational evidence for PARPi monotherapy and PARPi-based combinations across disease states. In first-line metastatic castration-resistant prostate cancer (mCRPC), PARPi plus ARPI consistently prolongs radiographic progression-free survival, with the greatest benefit in HRR-altered tumors, and emerging overall-survival signals in selected subgroups. In later-line settings, monotherapy activity is most robust in BRCA2-mutated disease, whereas non-BRCA HRR alterations show heterogeneous and often modest responses, underscoring the need for biomarkers beyond gene panels. We also discuss combination strategies with DDR-targeting agents, radioligand therapies, and immunotherapy, and summarize ongoing phase III programs in metastatic castration-sensitive prostate cancer (mCSPC). Finally, we outline practical considerations for biomarker-informed patient selection, monitoring, sequencing, and toxicity management, with particular emphasis on intercepting MRD and resistance evolution.

## 1. Introduction

Prostate cancer is one of the most frequently diagnosed malignancies and a leading cause of cancer-related death in men worldwide [1]. Despite effective local therapy for organ-confined disease, a considerable proportion of patients either present with de novo metastatic disease or eventually relapse after initial curative intent treatment [1]. Androgen deprivation therapy (ADT) remains the backbone of systemic management across disease stages, and the addition of docetaxel or next-generation androgen receptor pathway inhibitors (ARPIs) improves survival in metastatic castration-sensitive prostate cancer [2,3,4,5]. Once metastatic castration-resistant prostate cancer (mCRPC) develops, the disease remains incurable, and median overall survival is limited to only a few years, even with sequential use of taxanes, ARPIs, and radioligand therapies [6,7,8,9].

Comprehensive genomic profiling has revealed that pathogenic DDR gene alterations occur in approximately 20–25% of mCRPC cohorts, based largely on combined germline and somatic analyses, with BRCA1/2 alterations representing a sizeable subset [10,11]. Importantly, DDR is an umbrella term that includes HRR as one repair pathway; therefore, alterations in genes such as ATM or CDK12 should not be assumed to confer the same biology or PARP inhibitor sensitivity as canonical BRCA-associated HRR deficiency. This estimate varies with cohort composition and whether monoallelic alterations or variants of uncertain significance are included and therefore should not be equated with the prevalence of clinically actionable biallelic HRR deficiency. Across clinical studies, BRCA1/2 alterations, particularly BRCA2, are the most consistent biomarkers of benefit from PARP inhibition, whereas non-BRCA alterations are more heterogeneous, with limited activity in ATM-altered disease and a more variable signal in CDK12-altered tumors [10,11,12]. These molecular features provide a strong rationale for targeting DNA repair pathways in prostate cancer.

Poly(ADP-ribose) polymerases (PARP1 and PARP2) play central roles in the repair of single-strand DNA breaks. In tumors with HRR deficiency, pharmacologic PARP inhibition induces synthetic lethality by preventing repair of endogenous DNA damage and promoting replication fork collapse [11]. Preclinical studies have demonstrated bidirectional crosstalk between the androgen receptor (AR) pathway and the DDR. AR blockade downregulates HRR gene expression and increases reliance on PARP-mediated repair, whereas PARP inhibition can suppress AR signaling. These findings support the concept that co-inhibition of AR and PARP may enhance antitumor activity beyond HRR-deficient disease alone [13,14].

Based on this biology, several PARP inhibitors have been evaluated in mCRPC. Phase II–III trials established clinically meaningful activity of olaparib and rucaparib in men with HRR-altered mCRPC who had progressed on prior AR-targeted therapy, leading to regulatory approvals in this setting [10,12]. Across these and other studies, the greatest and most consistent benefit is observed in tumors with BRCA1/2 alterations, particularly BRCA2, whereas outcomes in non-BRCA HRR-associated genes are heterogeneous, with consistently limited activity in ATM-altered disease and a more variable, potentially meaningful signal in CDK12-altered tumors [10,11,12].

More recently, three large phase III trials, MAGNITUDE (niraparib plus abiraterone), PROpel (olaparib plus abiraterone), and TALAPRO-2 (talazoparib plus enzalutamide), evaluated first-line PARP inhibitor plus ARPI combinations in mCRPC [15,16,17]. All three studies demonstrated significant improvements in radiographic progression-free survival, with the greatest benefit observed in patients with tumors harboring HRR alterations, particularly BRCA1/2 [15,16,17]. Emerging overall survival signals and contemporary reviews indicate that PARP inhibitor-based combinations are becoming an important treatment option for appropriately selected patients with mCRPC [14,16,17]. PARP inhibitors are now being explored earlier in the disease course, including in metastatic castration-sensitive prostate cancer, and in combination with other DDR–targeted agents, radioligand therapies, and immunotherapies [11,14].

**Figure 1:**
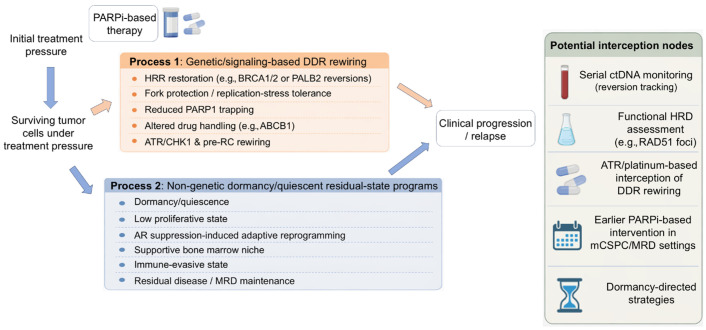
Dual-process model of PARPi resistance/resilience in prostate cancer and opportunities for interception. Under PARPi-based treatment pressure, surviving tumor cells may follow two parallel and potentially interacting routes that contribute to clinical progression or relapse. The first, shown in orange, consists of genetic and signaling-based DDR rewiring, including HRR restoration, fork protection/replication-stress tolerance, reduced PARP1 trapping, altered drug handling, and ATR/CHK1 or pre-RC rewiring. The second process, shown in blue, consists of non-genetic dormancy/quiescent residual-state programs, including low proliferative states, AR suppression-induced adaptive reprogramming, supportive bone marrow niches, immune-evasive states, and maintenance of minimal residual disease (MRD). These processes may coexist and converge to drive relapse. The left-side arrows indicate that both routes can emerge from surviving tumor cells under treatment pressure, whereas the right-side arrows indicate their contribution to clinical progression/relapse. Potential interception nodes include serial ctDNA monitoring for reversion tracking, functional HRD assessment, ATR/platinum-based interception of DDR rewiring, earlier PARPi-based intervention in mCSPC/MRD settings, and dormancy-directed strategies.

We propose that resistance to PARPi-based therapy in prostate cancer may be usefully conceptualized as two convergent processes: (1) genetic and signaling-based DDR rewiring that restores HRR or buffers PARPi-induced replication stress, and (2) non-genetic adaptive persistence, including dormancy and quiescent residual tumor-cell states that maintain MRD under AR pathway suppression and PARP blockade. This framework helps explain why clinical benefit is strongest in tumors with functionally consequential, often biallelic BRCA2 loss, why monoallelic or biologically ambiguous HRR alterations may not confer equivalent sensitivity, why non-BRCA HRR alterations are heterogeneous, and why improvements in radiographic progression-free survival do not necessarily translate into a clear overall-survival benefit, particularly in biologically unselected populations. This dual-process framework is summarized schematically in Figure 1.

In this review, we use the term “resistance” broadly to refer to processes that reduce treatment efficacy, while recognizing that some non-genomic dormant/quiescent states may be more accurately described as adaptive persistence or resilience rather than classical resistance. Guided by the dual-process framework summarized in Figure 1, we examine the evolving role of PARP inhibitors in prostate cancer, with an emphasis on PARPi monotherapy and PARPi combinations with ADT and ARPIs. We discuss clinical efficacy by molecular subgroup, practical considerations for biomarker testing and patient selection, timing and sequencing strategies, and toxicity management. We also integrate emerging resistance mechanisms, including DDR rewiring and treatment-induced dormancy/quiescent residual states, to highlight actionable opportunities to intercept MRD and delay relapse after PARP- and AR-targeted therapies.

## 2. Mechanisms of Action of PARP Inhibitors in Prostate Cancer

**Figure 2:**
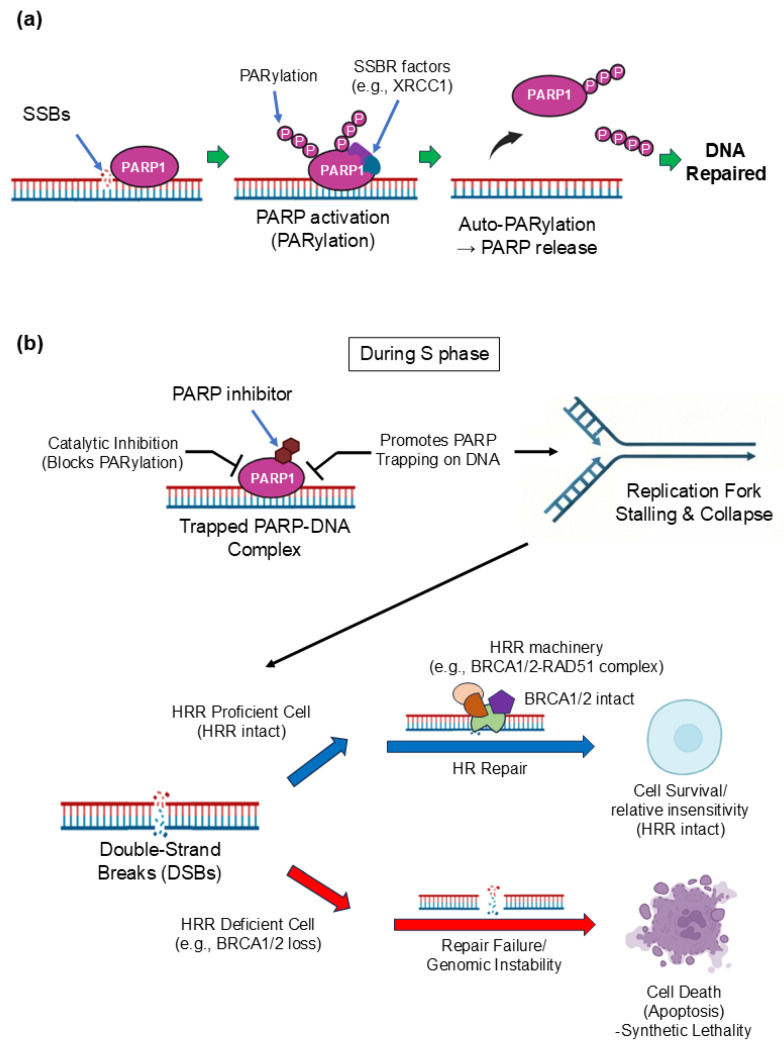
Mechanisms of action of PARP inhibitors. (**a**) Normal PARP Function & SSBR Pathway: PARP1 recognizes DNA SSBs and catalyzes PARylation, recruiting single-strand break repair (SSBR) factors (e.g., XRCC1) and facilitating repair and PARP1 release. In panel (**a**), black arrows indicate the sequence of events, including auto-PARylation followed by PARP1 release from DNA. (**b**) PARP inhibition: Catalytic Blockage & Trapping leading to Synthetic lethality or Resistance: PARP inhibitors block PARylation and stabilize PARP1–DNA complexes (PARP1 trapping). When replication forks encounter trapped PARP1 or unrepaired lesions, forks stall/collapse, generating DSBs. DSBs can be repaired by homologous recombination repair (HRR) in HRR-proficient cells (e.g., BRCA1/2-intact). In contrast, HRR-deficient cells (e.g., BRCA1/2-loss) fail to repair DSBs, leading to genomic instability and apoptosis (synthetic lethality). PARP1-trapping potency differs among clinically used PARP inhibitors. The colored schematic symbols represent HRR machinery, as indicated in the figure. Abbreviation: single-strand breaks (SSBs), single-strand break repair (SSBR), double-strand breaks (DSBs), homologous recombination repair (HRR). Created with BioRender.com.

PARP1 and PARP2 are rapidly recruited to DNA single-strand breaks (SSBs) and catalyze poly(ADP-ribose)ation, generating PAR chains that help recruit and organize SSB repair factors such as XRCC1 and the DNA ligase III complex [18]. PARP inhibitors block this catalytic activity, allowing SSBs and associated repair intermediates to persist and, during DNA replication, be converted into more toxic lesions, including replication-associated double-strand breaks (DSBs) that typically require homologous recombination repair (HRR) for resolution [19,20]. Accordingly, HRR-defective cells, particularly those with BRCA1/2 loss, are exquisitely sensitive to PARP inhibition through a synthetic lethal interaction leading to chromosomal instability and apoptosis [19,20]. Beyond catalytic inhibition, many clinically relevant PARP inhibitors also trap PARP1/2 on DNA at damage sites, forming stabilized PARP–DNA complexes that impede replication fork progression and amplify replication stress. Trapping potency varies among agents and can contribute to differences in cytotoxicity [21,22]. Recent work further suggests that PARP inhibition can provoke additional HRR-dependent DNA damage through mechanisms such as transcription–replication conflicts, refining classical models of PARP inhibitor lethality [23]. Finally, unresolved DNA damage under PARP inhibition may lead to cytosolic DNA accumulation and activate innate immune sensing via the cGAS–STING pathway, providing a biological rationale for ongoing efforts to combine PARP inhibitors with immunotherapy [24]. These canonical and emerging mechanisms of PARP inhibitor action are summarized schematically in Figure 2.

## 3. Molecular Mechanisms of PARP Inhibitor Resistance in Prostate Cancer

Despite meaningful activity of PARP inhibitors in a subset of metastatic prostate cancers with HRR alterations, resistance eventually emerges in most patients who initially respond. Conceptually, mechanisms of resistance can be grouped into those that (i) restore HRR capacity, (ii) limit replication-associated lethality by stabilizing stalled replication forks, (iii) reduce PARP1 trapping and effective target engagement, (iv) decrease intracellular drug exposure through altered drug transport or metabolism, or (v) rewire DDR and replication programs to tolerate PARP inhibitor-induced stress better. In addition to genetic routes that reconstitute HRR or buffer replication stress, PARPi resistance likely also involves non-genetic adaptive states, including dormancy and quiescent residual tumor-cell states that sustain minimal residual disease (MRD) under AR pathway suppression and PARP blockade and may contribute to relapse. These adaptive states are discussed in detail in Section 6 but are introduced here as a central component of resistance that may coexist with genetic mechanisms within the same patient. These mechanisms are not mutually exclusive and may coexist within the same patient, often in a polyclonal pattern detectable by serial analyses of tumor tissue or circulating tumor DNA.

### 3.1. HRR Restoration: BRCA1/2 (And PALB2) Reversion and Functional Recovery

Restoration of HRR function is one of the best-characterized and clinically important routes to acquired resistance in BRCA1/2- or PALB2-altered prostate cancer. Secondary “reversion” events can remove or bypass the original deleterious lesion (for example, compensatory insertions or deletions that restore the open reading frame), leading to re-expression of functional BRCA1/2 or PALB2 and, consequently, renewed HRR proficiency. Serial circulating tumor DNA (ctDNA) profiling from TOPARP-A showed that, after an initial response to olaparib, multiple subclonal events could emerge that restored BRCA2 or PALB2 alterations to an in-frame configuration, supporting reversion as a clinically relevant resistance mechanism in mCRPC [25]. Case-level clinical observations have similarly documented biallelic BRCA2 reversion mutations coincident with acquired olaparib resistance [26]. In TRITON2, plasma-based analyses also identified BRCA reversion mutations after progression on rucaparib, frequently showing subclonal convergence suggestive of systemic therapeutic selection pressure [27].

Recent analyses of paired samples have sharpened mechanistic insight into how reversions arise. In TOPARP-B specimens, reversion mutations were detected in most BRCA2/PALB2-mutated tumors by the end of treatment (79%), and among reversions mediated by frameshift deletions, microhomology at breakpoints was common (60%), implicating polymerase theta (POLQ)-associated microhomology-mediated end joining as a plausible generator of clinically observed reversions [28]. Both the number of distinct reversion events and their timing were associated with radiographic progression-free survival and overall survival, suggesting that the tempo and polyclonality of reversion evolution may carry prognostic information [28]. Together, these data support HRR restoration, especially via reversion, as a dominant acquired mechanism that can be tracked by liquid biopsy during PARP inhibitor treatment.

HRR recovery can also be assessed functionally. RAD51 nuclear foci formation, measured by immunofluorescence, is a practical readout of HRR competence and can help distinguish tumors with true functional HRR deficiency from those with genomic “DDR alterations” but preserved HRR activity. In TOPARP-B, low RAD51 foci largely correlated with biallelic BRCA1/2 or PALB2 loss, whereas many ATM- and CDK12-altered tumors retained higher RAD51 foci levels, consistent with less pronounced functional HRR deficiency [29]. Across tumor types, RAD51 foci assays have also been linked to PARP inhibitor resistance in germline BRCA-mutated disease, supporting the broader idea that recovery of RAD51 foci can mark functional HRR restoration under selective pressure [30].

### 3.2. Replication Fork Stabilization: Limiting MRE11-Mediated Degradation and Related Pathways

Replication fork stabilization (fork protection) represents a distinct resistance route that can operate even without full HRR restoration. In HRR-defective cells, PARP inhibitors can amplify replication stress, promote fork stalling, and increase the likelihood of fork collapse and genome instability. A key vulnerability in BRCA-deficient states is excessive nuclease-mediated degradation of stalled forks, particularly by MRE11, which contributes to cytotoxicity [31,32]. Multiple genetic or epigenetic adaptations that reduce MRE11 recruitment or activity at stalled forks can therefore diminish the cytotoxic effects of PARP inhibition.

Several pathways modulate fork protection. The Fanconi anemia pathway, including FANCD2 have repair-independent roles in protecting stalled forks from degradation and interfaces with RAD51-BRCA1/2–dependent fork stability [33]. Chromatin and fork-associated factors such as PTIP influence MRE11 recruitment; loss of PTIP limits MRE11 loading at stalled forks and improves survival of BRCA-deficient cells under genotoxic stress, in experimental systems [34]. Consistent with this mechanism, limiting MRE11 accumulation at stalled forks can mitigate fork degradation and reduce the lethal consequences of PARP inhibition in BRCA2-deficient models [32]. At present, evidence for fork protection as a PARP inhibitor resistance mechanism in prostate cancer is strongest in preclinical BRCA-deficient systems, whereas direct clinical validation in mCRPC, including paired biopsy or ctDNA-based correlates, remains limited. In prostate cancer, this category may be especially relevant when HRR restoration through BRCA reversion does not occur, allowing tumor survival by shifting from repair restoration toward fork tolerance programs.

### 3.3. PARP1 and PARP Trapping: Target Alteration and Reduced Cytotoxic Lesions

PARP inhibitor cytotoxicity is closely tied to PARP1 trapping on chromatin, which can physically obstruct replication and convert replication-associated lesions into lethal DNA damage. Resistance can arise when tumors reduce PARP1 trapping or otherwise diminish effective PARP1 engagement. A genome-wide CRISPR “tag-mutate-enrich” screen identified multiple PARP1 mutations that confer resistance to PARP inhibitors by altering PARP1 DNA binding and/or trapping; notably, a clinically observed PARP1 mutation (R591C) was linked to reduced trapping and resistance [35]. Although clinically observed PARP1 variants support biological plausibility, current evidence in mCRPC remains limited, and PARP1 target alteration should presently be regarded as an emerging, likely uncommon acquired resistance mechanism that requires longitudinal clinical validation. These data reinforce that PARP1 itself can be an acquired resistance locus and suggest that the resulting resistance genotype may shape cross-resistance patterns and subsequent therapeutic vulnerabilities.

### 3.4. Drug Transport Programs: ABCB1 and Reduced Intracellular Drug Exposure

A more pharmacologic route to resistance involves reduced intracellular exposure to PARP inhibitors. In advanced prostate cancer models, ABCB1 (MDR1) upregulation mediated cross-resistance between taxanes and olaparib, and this cross-resistance could be reversed by genetic or pharmacologic suppression of ABCB1 [36]. ABCB1-mediated reduced drug exposure is supported by advanced prostate cancer model systems and may help explain treatment-sequence effects, but direct prospective clinical evidence linking ABCB1 upregulation to PARP inhibitor failure in mCRPC is still limited. This observation offers a mechanistic explanation for treatment-sequence effects in ARPI- and taxane-exposed tumors and highlights that resistance can be driven by a drug-handling phenotype rather than by DNA repair rewiring.

### 3.5. DDR Network Rewiring (CHEK2, ATR/CHK1) and Replication Initiation Programs (Pre-RC)

Under PARP inhibition, tumors may increasingly depend on replication stress checkpoints and alternative DDR signaling to survive. The ATR-CHK1 axis is a central coordinator of replication stress tolerance. Experimentally, SLFN11 loss is a strong determinant of PARP inhibitor resistance and creates reliance on ATR signaling, so that ATR inhibition can overcome resistance in SLFN11-deficient settings [37]. More broadly, ATR inhibition has been shown to disrupt rewired HRR and fork protection programs in PARP inhibitor-resistant BRCA-deficient cells, providing a mechanistic rationale for PARP inhibitors plus ATR inhibitor strategies in resistant states [38].

Prostate cancer-focused functional genomics is also revealing context-specific DDR wiring. A CRISPR screen in prostate cancer models reported that CHEK2 loss can induce PARP inhibitor resistance, at least in part by increasing BRCA2 expression via CHEK2–TP53–E2F7 circuitry; importantly, this resistance was mitigated by combined ATR and PARP inhibition [39]. These findings connect a clinically frequent DDR gene (CHEK2) to a plausible resistance phenotype that does not require BRCA reversion and again point to ATR as a rational interception node.

Finally, replication initiation and licensing programs have emerged as an unexpected determinant of PARP inhibitor response. In murine BRCA2-deficient prostate organoids engineered to preclude reversion, a genome-wide CRISPR screen identified multiple pre-replication complex (pre-RC) genes (CDT1, CDC6, DBF4) whose depletion conferred resistance to olaparib and to the PARP1-selective inhibitor AZD5305 [40]. Mechanistically, impaired pre-RC activity accelerated the resolution of olaparib-induced DNA damage and protected nascent DNA from fork degradation in BRCA2-deficient cells. Notably, in the CRPC genomic cohort analyzed by Pappas et al. [40], copy-number loss of pre-RC genes, particularly CDT1, was observed in approximately half of tumors; however, this was a cross-sectional genomic observation rather than an outcome-linked biomarker analysis, and a direct association with PARP inhibitor resistance in BRCA2-mutant mCRPC remains to be established. Collectively, the emerging picture is that PARP inhibitor resistance in prostate cancer can be achieved by restoring HRR, preventing replication-associated damage from becoming lethal, or reducing effective drug-target interactions, with significant opportunities for biomarker-driven interception. Thus, the pre-RC signal currently rests on prostate organoid screening with supportive cross-sectional CRPC genomic data, but a direct clinical correlation with PARP inhibitor outcome in BRCA2-mutant mCRPC has not yet been demonstrated.

### 3.6. Dormancy and Quiescent Residual States as a Putative Non-Genetic Process of Resistance and Viable MRD Maintenance

Dormancy and quiescent therapy-tolerant residual states may represent a complementary, non-genetic contributor to PARP inhibitor resistance and MRD maintenance [41,42]. More generally, because PARP inhibitor cytotoxicity is tightly linked to replication-associated DNA damage, reduced proliferation or quiescence is biologically expected to lessen S-phase-linked lesions and thereby attenuate vulnerability to PARP inhibition [21,43]. In prostate cancer specifically, androgen deprivation and AR pathway suppression can induce dormancy-associated states with coordinated downregulation of proliferation and DNA repair programs [44,45,46]. These states may be further supported by protective bone and immune microenvironments, as discussed further in Section 6 [47,48,49]. However, direct evidence that these states causally reduce PARP inhibitor sensitivity in patients with prostate cancer, particularly under combined AR and PARP blockade, remains limited. We therefore present dormancy/quiescence as a biologically plausible, prostate-relevant resistance framework that warrants direct testing in longitudinal MRD-focused studies and dormancy-capable prostate cancer models.

## 4. Crosstalk Between AR Signaling and the DNA Damage Response: How AR Inhibition Reshapes DNA Repair Phenotypes and Supports PARPi-Based Double and Triple Combinations

A growing body of work supports a functional coupling between AR signaling and the DDR in prostate cancer. AR activity is not only a driver of tumor growth, but also a regulator of DNA repair capacity, and AR pathway inhibition can alter the balance between HRR, non-homologous end joining (NHEJ), and compensatory repair programs. This convergence provides a mechanistic framework for combining PARP inhibitors with ADT and an ARPI to induce repair vulnerability and intensify replication-associated DNA damage [50,51,52,53].

### 4.1. AR as a Transcriptional Regulator of DNA Repair Genes

In prostate cancer, androgen receptor (AR) signaling transcriptionally upregulates a broad network of DNA repair genes, and high AR transcriptional output is associated with increased expression of DNA repair programs in both clinical specimens and experimental models. Androgen stimulation enhances repair of DNA damage after ionizing radiation, whereas treatment with second-generation antiandrogens downregulates DNA repair gene expression, increases DNA damage, and reduces clonogenic survival. Notably, AR pathway inhibition also reduces classical NHEJ activity, indicating that AR can influence multiple repair pathways beyond homologous recombination repair (HRR) [51]. Consistent with these observations, preclinical work has described a hormone-DNA repair circuit in which genotoxic stress can feed back to reinforce AR signaling. At the same time, androgens promote double-strand break repair and resistance to genotoxic insult. Together, these studies support a model in which AR signaling actively shapes the DNA repair landscape of prostate tumors, and suppression of AR creates a state of heightened vulnerability to DNA damage [50,51].

### 4.2. AR Inhibition Induces a Functional HRR-Deficient (“BRCAness”) Phenotype and Increases PARPi Sensitivity

Several studies have provided direct evidence that ADT or ARPI can functionally impair HRR and increase reliance on PARP-dependent repair. In prostate cancer cells, intact AR signaling is required to maintain HR gene expression and HR function; suppression of the AR pathway reduces HR capacity and is accompanied by upregulation of PARP-mediated repair pathways after ADT, supporting the idea that ADT can induce a functional HRR-deficient state even before overt castration resistance develops [52]. These data provide a mechanistic basis for therapeutic synthetic lethality by combining AR suppression with PARP inhibition. In parallel, work in BRCA-wild-type castration-resistant models has shown that enzalutamide downregulates HR-associated genes and induces a “BRCAness” phenotype. That sequential AR inhibition followed by PARP inhibition can produce synthetic lethality in vitro and in vivo [53]. Together, these findings support ARPI-induced HRR dysfunction as a strategy to broaden PARP inhibitor sensitivity beyond tumors with canonical HRR gene mutations.

### 4.3. PARP Enzymes as Facilitators of AR and AR-Variant Transcriptional Programs

The coupling between AR signaling and PARP biology appears bidirectional. In AR-positive prostate cancer models, PARP1 has context-dependent transcriptional roles that can support tumor growth and progression to castration resistance [54]. Mechanistic work in HRR-wild-type models suggests that the activity of ARPI plus PARP inhibition depends on ARPI-responsive tumor states and inhibitor-specific PARP1-trapping potency, and that PARP1 can modulate AR recruitment to chromatin under DNA damage [55]. Evidence also links AR splice variants to PARP biology: AR-V7 has been reported to enhance DNA damage response programs and upregulate DNA repair genes [56,57]. In clinical specimens and model systems, AR splice variant expression correlates with increased DNA repair gene expression and may contribute to reduced benefit from adding PARP inhibition in some settings [57]. PARP2 has also been implicated as a component of AR transcriptional machinery through interaction with the pioneer factor FOXA1, and recent work reinforces the central role of FOXA1 in enabling AR signaling [58,59]. Collectively, these findings suggest that combining PARP inhibitors with ARPIs may impose dual pressure through DNA damage intensification and perturbation of AR-driven programs, although the relative contribution of these components is likely to vary across tumor contexts [54,55,56,57,58,59].

### 4.4. Mechanistic Basis for ARPI-PARPi Triplet Combinations (PARPi + ARPI + ADT)

Preclinical combination studies demonstrate synergy between ARPIs and PARP inhibitors and highlight heterogeneity in the underlying drivers of benefit. In AR-positive castration-resistant prostate cancer models, enzalutamide plus olaparib synergistically suppresses cell growth, increases apoptosis, and enhances DNA damage; transcriptomic profiling suggests that these combination effects involve multiple pathways, including partial inhibition of non-homologous end joining via downregulation of DNA-PKcs and XRCC4, indicating that mechanisms beyond HRR suppression alone may contribute to synergy [60]. More recently, prostate cancer models lacking HRR mutations have been used to clarify why ARPI plus PARP inhibition remains effective in the absence of canonical HRR defects. In these systems, combination benefit requires ARPI-responsive cells and a PARP inhibitor with strong PARP1-trapping activity, and is driven by increased double-strand breaks and micronuclei formation; notably, the combination effect does not depend on PARP inhibitor-mediated suppression of AR transcription, and PARP1 appears to modulate AR recruitment to chromatin in the setting of DNA damage [55].

These observations align with seminal preclinical evidence supporting AR–PARP synthetic lethality [52] and are also summarized in recent reviews integrating the expanding preclinical and clinical data [61,62]. Taken together, they provide a coherent biological rationale for PARP inhibitor-based double and triple combinations. ADT plus an ARPI can reduce HRR gene expression and HR function in AR-driven tumors, creating a functional HRR-deficient state and increasing reliance on PARP-mediated repair, and adding a PARP inhibitor can then convert replication-associated lesions into lethal DNA damage, particularly when PARP1 trapping is robust [55], with classic mechanistic support for ARPI-induced HRR impairment from earlier work [52,53]. This framework also suggests practical boundaries: the benefit of a PARP inhibitor plus ARPI is expected to be greatest in tumors that remain AR-dependent and may depend on agent-specific pharmacology, including PARP1-trapping potency [55].

## 5. Clinical Evidence for PARP Inhibitors in Prostate Cancer: Monotherapy and Combination Strategies

Across trials, PARPi benefit appears to scale with functional homologous recombination deficiency rather than the mere presence of any DDR alteration, with BRCA2 loss representing the most consistent sensitivity state. By contrast, several non-BRCA “HRR genes” (e.g., ATM, CDK12, CHEK2) often retain RAD51 function and show heterogeneous phenotypes, which likely underlie variable clinical benefit. Therefore, clinical outcomes can be interpreted through a biology-first lens integrating the degree of HRD, AR dependence, replication-stress context, and the capacity to enter dormant/quiescent residual states.

### 5.1. PARP Inhibitor Monotherapy in mCRPC

Clinical evidence supports PARP inhibitor monotherapy in biomarker-selected mCRPC, with BRCA1/2 alterations, especially BRCA2 loss, emerging as the most reliable predictors of benefit. Biomarker-enriched phase II olaparib studies (TOPARP-A and TOPARP-B) first showed that responses cluster in tumors with deleterious DNA damage response defects and are most frequent and durable in BRCA1/2-altered disease [63,64]. PROfound provided randomized phase III confirmation by demonstrating longer imaging-based progression-free survival with olaparib compared with the physician’s choice of abiraterone or enzalutamide after progression on an ARPI, and an overall survival advantage despite substantial crossover [10,65]. Rucaparib also demonstrated meaningful radiographic and PSA responses in BRCA1/2-altered mCRPC (TRITON2) and confirmatory benefit versus physician’s choice therapy in TRITON3 [12,66]. Additional phase II programs have supported activity for talazoparib (TALAPRO-1) and niraparib (GALAHAD) in heavily pretreated DDR/HRR-altered populations [67,68]. Across studies, responses in non-BRCA alterations are more heterogeneous, with consistently limited activity in ATM- and CHEK2-altered disease and a more variable, generally modest signal in CDK12-altered tumors, highlighting the need for refined biomarkers and motivating combination and earlier-line strategies [69].

### 5.2. Phase III Evidence in First-Line mCRPC

In first-line mCRPC, multiple randomized phase III trials have tested whether adding a PARP inhibitor to an ARPI can extend benefit beyond biomarker-selected populations. We summarize the design and key outcomes of the major phase III trials in Table 1.

PROpel evaluated first-line mCRPC using PARPi plus AR pathway inhibition in an all-comers population and demonstrated a consistent improvement in radiographic progression-free survival [70]. However, at the final prespecified overall-survival analysis, median OS was numerically longer with olaparib plus abiraterone (42.1 vs. 34.7 months; HR 0.81), but the result did not cross the prespecified threshold for statistical significance (*p* = 0.054) [16]. These findings support a biology-first interpretation in which patient selection informed by functional HRD, rather than broad eligibility alone, may better enrich for durable benefit [29].

MAGNITUDE tested PARPi plus AR pathway inhibition with prospective stratification by HRR alteration status and revealed a clear contrast between biomarker-defined subgroups [15]. The lack of meaningful benefit in HRR-negative disease underscores that the presence or absence of a labeled “HRR gene alteration” is an imperfect surrogate for true, functional HRD [71]. Collectively, the trial reinforces the need for biomarkers that capture HRD biology and resistance liability beyond gene panels [29].

TALAPRO-2 evaluated PARPi plus ARPI in first-line mCRPC and demonstrated a robust delay in radiographic progression; final overall-survival analysis in the all-comers cohort was also positive [72]. However, the magnitude of OS benefit was greater in HRR-deficient disease than in HRR-non-deficient/unknown disease, with particularly strong benefit in BRCA-centered biology [72]. These findings support biomarker-guided intensification and further motivate efforts to refine subgroup definitions using functional readouts such as RAD51 competence [30] and dynamic ctDNA monitoring [25,27,73].

Collectively, these trials establish ARPI-PARPi triplets as effective rPFS-prolonging strategies in first-line mCRPC, whereas OS interpretation remains regimen- and biomarker-context dependent: PROpel showed a numerically favorable but statistically non-significant final OS result in an unselected population, MAGNITUDE showed no significant univariate OS difference in HRR-positive disease at final analysis, and TALAPRO-2 demonstrated significant OS benefit with stronger effect in HRR-deficient/BRCA-centered subsets [15,16,17,70,71,72,74].

### 5.3. Practical Framework for Biomarker Testing, Patient Selection, and Current FDA-Labeled Use of PARP Inhibitors

Because the clinical question is no longer simply whether a tumor is “HRR-altered,” biomarker-guided use of PARP inhibitor-based therapy should be organized around three practical decision points: (1) label-defining eligibility testing, (2) biology-refining assessment of likely benefit, and (3) longitudinal molecular surveillance for acquired resistance. Table 2 summarizes a pragmatic framework integrating these steps with current FDA-labeled use in prostate cancer.

In practice, germline and tumor-based testing establish whether a currently available PARPi strategy is relevant, whereas deeper interpretation of BRCA-centered biology, biallelic status, and, where feasible, functional HRD assays may refine expected benefit, particularly in non-BRCA disease [29,30]. Serial ctDNA can complement tissue-based assessment by tracking reversion-mediated escape and informing sequencing at progression [25,27,73].

A concise clinical workflow organizing PARP inhibitor use around label-defining eligibility testing, benefit-refining biological assessment, and resistance-tracking surveillance. Current FDA-labeled entries are based on the current U.S. prescribing information for Lynparza, Talzenna, Akeega, and Rubraca [75,76,77,78].

### 5.4. Expansion into mCSPC: Ongoing and Emerging Phase III Programs

Building on mCRPC data, multiple phase III programs are evaluating PARPi–ARPI strategies earlier in metastatic castration-sensitive prostate cancer (mCSPC). An overview of the ongoing phase III programs is provided in Table 3.

TALAPRO-3 is a randomized phase III study assessing talazoparib plus enzalutamide in men with HRR gene-altered mCSPC receiving androgen deprivation therapy, designed to test whether earlier use of PARP–AR co-targeting can delay radiographic progression and improve survival in a biomarker-enriched population [80].

AMPLITUDE reported that adding niraparib to abiraterone/prednisone plus ADT improved rPFS in HRR-altered mCSPC, while overall-survival data remain immature, supporting earlier biomarker-enriched intensification and the need for refined functional HRD stratification beyond gene panels [79].

EvoPAR-Prostate01 is a large phase III trial testing saruparib (AZD5305), a next-generation PARP1-selective inhibitor and trapper, in combination with the physician’s choice ARPI (abiraterone, enzalutamide, or darolutamide) in two parallel cohorts defined by HRRm status (HRRm and non-HRRm), with rPFS as the primary endpoint within each cohort (ClinicalTrials.gov NCT06120491) [81,82].

This design will help clarify whether a more selective PARP1 inhibitor can widen the therapeutic window and potentially extend combination benefit beyond classical HRR-mutant settings while preserving tolerability [81,82] (Table 3).

## 6. Treatment-Induced Dormancy, Bone Marrow Niches, and the Rationale for Early PARP Inhibitor-Based Combinations

Treatment-induced tumor dormancy is increasingly recognized as a distinct phase of cancer evolution in which residual disease persists in a clinically silent, non- or slowly proliferative state, yet retains the capacity to drive late relapse and metastasis once suppressive pressures are lifted or bypassed [83,84,85]. In prostate cancer, ADT often induces a prolonged period of biochemical and radiographic quiescence, best understood as an ADT-induced dormancy state rather than the true eradication of malignant cells [44,86]. Work from patient-derived xenograft (PDX) and organoid models has shown that residual prostate cancer cells under chronic androgen suppression undergo coordinated transcriptional reprogramming of cell cycle, DNA repair, and stress-response programs, supporting the concept that dormancy is an active, plastic adaptation to therapy rather than a purely passive G0 arrest [44,45,46].

ADT–induced dormancy has been modeled in detail using hormone-naïve prostate cancer PDXs [44]. In these systems, castration of mice bearing hormone-sensitive PDXs generates multiple castration-induced dormant prostate cancer models that closely recapitulate the clinical course after ADT, and two major dormancy subtypes have been described—cellular dormancy and tumor-mass dormancy—with distinct morphology, transcriptomic features, and relapse kinetics [44]. A dormancy-subtype-based predisposed gene signature derived from pre-castration tumors predicts response to neoadjuvant ADT in hormone-naïve prostate cancer and clinical outcome in castration-resistant disease treated with ADT or AR pathway inhibitors, underscoring that dormancy behavior is at least partly “pre-wired” in the primary tumor [44]. Complementary studies using the same PDX platforms have reconstructed the intrinsic immune landscape of castration-induced dormant prostate cancer, revealing immune-evasion programs in dormant tissue and highlighting dormancy as a state in which tumor cells must actively evade immune surveillance to persist in bone and renal-capsule niches [48].

At the cellular level, these and other studies support the idea of “dormancy-capable cells”—a subset of cancer cells endowed with the ability to cycle between quiescence and proliferation, and to survive in protective niches for extended periods [48,83,84,86]. Crea et al. proposed that such dormancy competent cancer cells are driven less by fixed genetic changes and more by reversible epigenetic and non-coding RNA circuits, providing a conceptual bridge between cancer stem-like properties, treatment resistance, and dormancy plasticity [84]. In PDX models of ADT-induced dormancy, dormant lesions retain AR expression but exhibit broad downregulation of proliferation and DNA repair genes, together with subtype-specific differences in pathways such as Wnt/β-catenin, apoptosis, p38 MAPK, integrin, and epithelial–mesenchymal transition signaling, all of which may shape whether tumors enter cellular versus tumor mass dormancy and how quickly they eventually relapse [44].

Recent work has further linked dormancy to specific microenvironmental and immune niches. Using PDX models that reliably enter ADT-induced dormancy, investigators have mapped the intrinsic immune landscape of dormant tumors and identified immune-modulatory gene programs that enable dormant prostate cancer cells to evade T cell- and NK cell-mediated clearance [48]. Single-cell and bulk RNA-sequencing studies have also implicated immune checkpoint and B7 family ligands in this process, including B7-H4, which is selectively upregulated during ADT-induced dormancy and functionally supports survival of low-proliferative cells within an extracellular matrix–rich niche [49]. In functional assays, B7-H4 expression in LNCaP cells reduced proliferation under androgen-deprived conditions and delayed relapse in castrated hosts, suggesting that a subset of dormancy-capable cells may depend on specific ECM–receptor interactions and immune-evasion programs to maintain long-term viability [49]. Other studies have begun to explore how exploiting the “pre-dormancy” immune microenvironment created by early ADT, before complete dormancy is established, might allow rational immunomodulatory combinations to eliminate these cells more effectively [87].

Conceptually, these data raise the possibility that PARP inhibitor-based combinations could be leveraged not only to treat overt HRR-deficient castration-resistant disease but also to target dormancy-capable cells before they fully adapt to chronic androgen suppression. ADT and ARPIs can reduce expression of HRR genes and induce a functional HRR-deficient state in AR-driven tumors, thereby increasing reliance on PARP-mediated repair and magnifying the impact of PARP inhibition on replication-associated DNA lesions [52,53]. Coupling ADT/ARPI with PARP inhibitors at a time when micrometastatic disease is still relatively proliferative, genomically stressed, and not yet fully embedded in protective bone and immune niches could, in theory, reduce the pool of dormancy-capable cells that later seed lethal castration-resistant relapses. Testing this hypothesis will require integration of established PDX and organoid dormancy platforms [44,45,46,48,49,87] with PARP inhibitor and ARPI combinations in the hormone-naïve setting, longitudinal sampling of minimal residual disease, and functional assays that directly quantify the impact of early combination therapy on dormancy initiation, maintenance, and reactivation.

## 7. Future Directions: Optimizing Patient Selection, Timing, and Resistance Management

Extensive preclinical work has shown that combining androgen receptor pathway inhibition with PARP inhibition can induce synthetic lethality even in models without canonical HRR gene mutations. However, in clinical practice, the most consistent and clinically meaningful benefit of PARP inhibitors, whether as monotherapy or in first-line mCRPC combinations, remains concentrated in tumors with BRCA1/2 alterations, particularly BRCA2 loss. In contrast, outcomes for ATM-altered disease are generally limited, whereas CDK12-altered tumors show more variable results, underscoring the biological heterogeneity of non-BRCA HRR-associated alterations [88]. This disconnect between robust preclinical synergy and more modest, gene-restricted benefit in patients represents a central clinical gap that future research must address [89].

One priority is to refine biomarker strategies beyond simple gene list-based HRR mutation panels. Current next-generation sequencing assays detect pathogenic alterations in genes such as BRCA1/2, ATM, PALB2, and CDK12, but they do not directly measure functional homologous recombination deficiency or capture the impact of co-occurring genomic alterations. Genomic scar scores and mutational signatures have been proposed as complementary tools to stratify PARP inhibitor sensitivity, and RAD51 foci-based functional assays, including immunohistochemistry-compatible protocols, are being developed and validated in other solid tumors as practical readouts of HRR competence [90]. Translating these functional HRD assays into prostate cancer and integrating them with gene-panel data will likely be essential to move from crude “HRRm versus non-HRRm” classifications toward more precise, biologically grounded predictors of who truly benefits from PARP inhibition [91]. Non-invasive molecular imaging may further complement tissue- and blood-based biomarker approaches for PARP inhibitor therapy. PARP-targeted PET radiotracers, such as [18F] FTT, offer a potential means to visualize PARP expression in vivo and to capture spatial heterogeneity that may not be fully reflected by static genomic testing alone. In a pilot study of advanced prostate cancer, FTT-PET/CT showed marked interpatient variability, higher uptake in HRR-mutated tumors, and potential utility as an alternative biomarker for PARP1 expression and PARPi treatment selection [92]. Although these approaches remain investigational, they may help refine biomarker-informed patient selection, pharmacodynamic assessment, and resistance monitoring in future prostate cancer studies.

Dynamic, treatment-integrated biomarkers will also be important for understanding and managing acquired resistance. Multiple studies have now shown that BRCA1/2 reversion mutations can emerge under selective pressure from PARP inhibitors or platinum chemotherapy in mCRPC, and that these events are detectable in serial circulating tumor DNA (ctDNA) well before or alongside clinical progression [27,73]. Routine ctDNA profiling in patients receiving PARP inhibitor-based therapy could therefore provide an early warning of emerging resistance, help distinguish actual progression from pseudo-progression or oligoprogression, and inform rational sequencing or combination strategies designed to suppress or delay reversion-mediated escape. Prospective trials embedding serial liquid-biopsy monitoring will be needed to define when and how such information should trigger therapy modification.

A second central area for future investigation is the earlier deployment of PARP inhibitor combinations in metastatic castration-sensitive prostate cancer. HRR alterations appear to occur early in the evolutionary history of many prostate tumors, and mCSPC is generally less genomically complex than heavily pretreated mCRPC. It is therefore plausible that targeting HRR-deficient clones with an ARPI plus a PARP inhibitor in the hormone-sensitive setting, when disease burden is lower, and tumor heterogeneity is less extreme, could yield larger and more durable benefits than when the same strategy is applied later. Ongoing phase III trials such as TALAPRO-3 and AMPLITUDE are directly testing PARP inhibitor–ARPI combinations in HRR-altered mCSPC [79,80], and EvoPAR-Prostate01 extends this paradigm by including a large non-HRR-mutated cohort, which will be critical for determining whether carefully dosed, PARP1-selective inhibition can safely broaden benefit to HRR wild-type disease [81,82]. In parallel, there is a strong rationale to examine how early PARP-based combinations interact with treatment-induced dormancy and bone-marrow niches, and whether they can reduce the pool of dormancy-capable cells that later seed lethal relapse [44,45,46,48,49,83,84,85,86,87,93].

In prostate cancer, autophagy is emerging as an additional determinant of PARP inhibitor response. In LNCaP, C4-2B, and PC-3 models, higher basal autophagy levels were associated with relative resistance to olaparib, and genetic depletion of autophagy via CRISPR/Cas9 knockout of ATG16L1 consistently increased olaparib sensitivity [94]. Pre-activation of autophagy with rapamycin before olaparib exposure reduced γH2AX accumulation and blunted the drug’s antiproliferative effect, whereas complete autophagy inhibition or autophagy activation after olaparib treatment enhanced olaparib-induced cytotoxicity [94]. Mechanistically, autophagy-mediated resistance was linked to reduced nuclear SQSTM1/p62, increased filamin A (FLNA), and enhanced recruitment of BRCA1 and RAD51 to DNA damage sites, promoting more efficient HR-mediated repair of olaparib-induced double-strand breaks [94]. Together with data from other tumor types showing that PARP inhibitors can induce predominantly cytoprotective autophagy in some contexts [95], these findings suggest that both basal autophagy status and the timing of autophagy modulation may influence PARP inhibitor sensitivity in prostate cancer and warrant systematic investigation in clinically relevant models.

Finally, the toxicity profile of current PARP inhibitors, particularly hematologic adverse events such as anemia and fatigue, underscores the need for better patient selection, not only a scientific challenge but also an urgent clinical need. As next-generation PARP1-selective agents advance and PARP inhibitor indications expand to earlier disease stages and combination regimens, future trials should embed robust translational programs that combine tumor tissue, organoid, and liquid biopsy studies with high-quality longitudinal clinical data. Such efforts will be crucial to define optimal timing and duration of PARP inhibitor-based therapy, to clarify which genomic and functional contexts truly justify combination intensification, and to determine whether early intervention can meaningfully alter the natural history of dormancy and resistance in advanced prostate cancer.

## 8. Conclusions

PARP inhibitors have expanded treatment options for advanced prostate cancer, with the most consistent clinical benefit observed in tumors harboring BRCA1/2 alterations, particularly BRCA2, both as monotherapy in mCRPC and as PARPi–ARPI combinations in first-line mCRPC. However, the benefit of non-BRCA HRR alterations and in HRR-wild-type disease remains variable, underscoring the need for biology-first patient selection beyond gene-panel status alone. In addition, both primary and acquired resistance commonly emerge through diverse mechanisms, including HRR restoration (e.g., BRCA reversions), replication-fork protection, altered drug handling, and broader DDR rewiring. Treatment-induced dormant/quiescent residual states, potentially supported by protective microenvironments such as bone marrow niches, may further sustain MRD and enable late relapse under AR- and PARP-targeted therapies.

Key priorities for the field include: (1) biomarkers beyond gene panels, integrating biallelic status, genomic scars/signatures, and functional assays such as RAD51 foci, and emerging imaging-based biomarkers such as PARP-targeted PET to define true HRD and refine patient selection; (2) dynamic monitoring with serial ctDNA and related approaches to detect reversion and polyclonal resistance early and guide sequencing; (3) earlier intervention, testing PARPi-based combinations in mCSPC and MRD settings where clonal complexity is lower and resistance evolution may be intercepted; and (4) dormancy-directed strategies that target persistence programs, niches, and immune evasion to eradicate MRD and delay relapse.

## Figures and Tables

**Table 1:** Phase III PARP inhibitor combinations on an ADT backbone in mCRPC.

Trial (Phase 3)	Disease State/Line	Population & Key Eligibility	HRR Selection	N (Randomized)	Experimental Regimen (on ADT Backbone)	Control Regimen (on ADT Backbone)	Primary Endpoint(s)	Key rPFS Result (Primary Analysis/Latest)	Key OS Result (Latest)	Key Publication(s)/Source URL(s)	NCT
PROpel	mCRPC; first-line (no prior life-prolonging therapy for mCRPC)	mCRPC; ECOG 0–1; prior docetaxel for mHSPC allowed; continued castration (orchiectomy or GnRH)	Unselected (all-comers); prespecified HRR/BRCA subgroup analyses	796 (399 vs. 397)	Olaparib + abiraterone + prednisone/prednisolone + ADT	Placebo + abiraterone + prednisone/prednisolone + ADT	Radiographic (imaging-based) PFS (rPFS/ibPFS)	24.8 vs. 16.6 mo; HR 0.66 (95% CI 0.54–0.81); *p* < 0.001	42.1 vs. 34.7 mo; HR 0.81 (95% CI 0.67–1.00, *p* = 0.054), not statistically significant.	Primary rPFS: https://pubmed.ncbi.nlm.nih.gov/38319800/ (accessed on 24 March 2026). Final OS: https://pubmed.ncbi.nlm.nih.gov/37714168/ (accessed on 24 March 2026).	NCT03732820
MAGNITUDE	mCRPC; first-line	Treatment-naïve mCRPC; continued castration; prospectively screened for HRR alterations; HRR− cohort stopped early for futility	Prospective HRR+ and HRR− cohorts; primary tested BRCA1/2 subgroup then overall HRR+	670 total (HRR+ 423; HRR− 247)	Niraparib + abiraterone + prednisone + ADT	Placebo + abiraterone + prednisone + ADT	rPFS (BRCA1/2 subgroup → HRR+ cohort); futility assessment in HRR− cohort	BRCA1/2: 16.6 vs. 10.9 mo; HR 0.53 (0.36–0.79); *p* = 0.001. HRR+: 16.5 vs. 13.7 mo; HR 0.73 (0.56–0.96); *p* = 0.022	Final OS (univariate): HRR+ HR 0.931 (0.720–1.203); *p* = 0.585. BRCA1/2 HR 0.788 (0.554–1.120); nominal *p* = 0.183 *	Primary rPFS: https://pubmed.ncbi.nlm.nih.gov/36952634/ (accessed on 24 March 2026). Final OS: https://pubmed.ncbi.nlm.nih.gov/40328571/ (accessed on 24 March 2026).	NCT03748641
TALAPRO-2	mCRPC; first-line	mCRPC on ADT; cohort 1 all-comers + cohort 2 HRR-deficient; randomized to talazoparib vs. placebo added to enzalutamide	All-comers with prospective HRR testing; coprimary rPFS in all-comers and HRR-altered populations	805 (cohort 1 all-comers); additional HRR-deficient cohort enrolled (combined HRR-deficient population reported separately)	Talazoparib + enzalutamide + ADT	Placebo + enzalutamide + ADT	rPFS by BICR (all-comers; HRR-altered population)	Primary (all-comers): NR vs. 21.9 mo; HR 0.63 (0.51–0.78); *p* < 0.0001. Updated: 33.1 vs. 19.5 mo; HR 0.67 (0.55–0.81); *p* < 0.0001	45.8 vs. 37.0 mo; HR 0.80 (0.66–0.96); *p* = 0.016 (final OS)	Primary rPFS: https://pubmed.ncbi.nlm.nih.gov/37285865/ (accessed on 24 March 2026). Final OS: https://pubmed.ncbi.nlm.nih.gov/40683290/ (accessed on 24 March 2026).	NCT03395197

Randomized phase III trials of PARP inhibitor plus AR pathway inhibition in first-line metastatic castration-resistant prostate cancer (mCRPC) on continued androgen deprivation therapy (ADT). Key eligibility and HRR selection, study arms, primary endpoint(s), and latest reported rPFS and OS outcomes are summarized. * Prespecified multivariate analyses adjusted for baseline prognostic factors showed more favorable trend estimates: HRR+ HR 0.785 (95% CI 0.606–1.016; nominal *p* = 0.066) and BRCA1/2 HR 0.663 (95% CI 0.464–0.947; nominal *p* = 0.024). Abbreviations: ADT, androgen deprivation therapy; HRR, homologous recombination repair; rPFS, radiographic progression-free survival; OS, overall survival; HR, hazard ratio; CI, confidence interval; BICR, blinded independent central review; ECOG, Eastern Cooperative Oncology Group; mHSPC, metastatic hormone-sensitive prostate cancer; GnRH, gonadotropin-releasing hormone; NR, not reached.

**Table 2:** Practical framework for biomarker testing, patient selection, and current FDA-labeled PARP inhibitor use in prostate cancer.

Clinical Setting/ Stage	Immediate Goal	Test(s) and Key Result(s)	Practical Implication/ Current FDA-Labeled Use	Monitoring/ Next Step
mCRPC after prior AR-targeted therapy	Determine eligibility for PARPi monotherapy	Germline testing + tumor NGS; plasma ctDNA if tissue is unavailable or inadequate. Prioritize BRCA1/2 versus other HRR genes; assess biallelic status if feasible.	Olaparib for HRR gene-mutated mCRPC after prior enzalutamide or abiraterone; rucaparib for BRCA-mutated mCRPC after prior AR-directed therapy.	Preserve the baseline molecular profile for later comparison; consider serial ctDNA if PARPi is started.
First-line mCRPC considering PARPi-ARPI combination	Match biomarker status to currently available doublets	Up-front germline + tumor NGS or validated plasma testing. Distinguish BRCA-mutated disease from broader HRR gene-mutated disease.	Olaparib + abiraterone or niraparib + abiraterone for BRCA-mutated mCRPC; talazoparib + enzalutamide for HRR gene-mutated mCRPC.	Use the baseline genomic profile as the reference for later resistance tracking.
Non-BRCA HRR alteration or equivocal biology	Refine likelihood of benefit beyond simple label eligibility	Biallelic assessment; genomic scars/signatures; functional HRD assays (e.g., RAD51), where available.	Not primarily label-defining, but useful for prioritizing expected benefit or trial enrollment in biologically ambiguous cases.	Integrate tissue and plasma data and re-evaluate at progression.
On-treatment or progression on PARPi-based therapy	Detect acquired resistance and guide sequencing	Serial ctDNA; repeat biopsy when feasible. Prioritize BRCA1/2 reversion and evolving polyclonal resistance.	Supports decisions about continuing PARP pressure versus switching, combining, or sequencing therapy differently.	Consider a baseline → on-treatment → progression sampling strategy.
mCSPC/early intensification	Identify patients for earlier biomarker-guided PARPi use	Up-front germline + tumor testing, with particular attention to BRCA status, especially BRCA2.	Akeega with prednisone is FDA-labeled for BRCA2-mutated mCSPC; broader HRR-defined use in mCSPC remains trial-driven.	Prospectively embed ctDNA/MRD studies where feasible.

Note: Label-defining tests establish whether a currently available PARPi strategy is applicable; benefit-refining tests help prioritize expected depth of benefit, especially in non-BRCA disease; resistance-tracking tests are used longitudinally to detect reversion-mediated escape and guide sequencing. Current FDA-labeled entries are based on the current U.S. prescribing information for Lynparza, Talzenna, Akeega, and Rubraca [75,76,77,78].

**Table 3:** Phase III PARP inhibitor combinations on an ADT backbone in mCSPC (mHSPC).

Trial (Phase 3)	Disease State/Line	Population & Key Eligibility	HRR Selection	N (Randomized)	Experimental Regimen (on ADT Backbone)	Control Regimen (on ADT Backbone)	Primary Endpoint(s)	Key rPFS Result (Primary Analysis/Latest)	Key OS Result (Latest)	Key Publication(s)/Source URL(s)	NCT
TALAPRO-3	mCSPC (mHSPC); biomarker-enriched	DDR/HRR gene–altered mCSPC receiving ADT; talazoparib vs. placebo added to enzalutamide	HRR gene alterations required	~550 planned	Talazoparib + enzalutamide + ADT	Placebo + enzalutamide + ADT	Investigator-assessed rPFS (RECIST 1.1/PCWG3); OS key secondary	Ongoing (no primary efficacy results reported yet)	Ongoing	Trial protocol: https://pubmed.ncbi.nlm.nih.gov/37882449/ (accessed on 24 March 2026).	NCT04821622
AMPLITUDE	mCSPC (mHSPC); biomarker-enriched	HRR gene–altered mCSPC; niraparib vs. placebo added to abiraterone acetate + prednisone, on ADT	HRR gene alterations required (hierarchical testing: BRCA subgroup → ITT)	696 (348 vs. 348)	Niraparib + abiraterone acetate + prednisone + ADT	Placebo + abiraterone acetate + prednisone + ADT	rPFS (hierarchical: BRCA subgroup then ITT); OS key secondary (immature at report)	BRCA subgroup: HR 0.52 (0.37–0.72); *p* < 0.0001. ITT: HR 0.63 (0.49–0.80); *p* = 0.0001	Immature; ITT HR 0.79 (0.59–1.04). BRCA HR 0.75 (0.51–1.11)	Primary publication: [79]	NCT04497844
EvoPAR-Prostate01	mCSPC (mHSPC); two-cohort design	mCSPC on ADT; two parallel cohorts: HRRm and non-HRRm; saruparib (AZD5305) vs. placebo added to physician’s choice ARPI	Cohort 1: HRRm; Cohort 2: non-HRRm (prospective tumor/ctDNA testing)	~1800 planned (≈550 HRRm; ≈1250 non-HRRm)	Saruparib (AZD5305) + physician’s choice ARPI (abiraterone/enzalutamide/darolutamide) + ADT	Placebo + physician’s choice ARPI + ADT	rPFS within each cohort; OS key secondary (within-cohort analyses)	Ongoing (no primary efficacy results reported yet)	Ongoing	ClinicalTrials.gov record: https://clinicaltrials.gov/study/NCT06120491 (accessed on 24 March 2026). ASCO abstract DOI: https://ascopubs.org/doi/10.1200/JCO.2025.43.5_suppl.TPS279 (accessed on 24 March 2026).	NCT06120491

Randomized phase III trials evaluating PARP inhibitor based intensification on an ADT backbone in metastatic castration-sensitive prostate cancer (mCSPC, also termed mHSPC), including biomarker-enriched and two-cohort designs. Key eligibility and HRR selection, study arms, primary endpoint(s), and current efficacy status for rPFS and OS are summarized. Note: Key genes (e.g., BRCA1/2, ATM) may be included in both DDR and HRR definitions. Abbreviations: DDR, DNA damage response; HRR, homologous recombination repair; ADT, androgen deprivation therapy; ARPI, androgen receptor pathway inhibitor; rPFS, radiographic progression-free survival; OS, overall survival; ITT, intention-to-treat; HR, hazard ratio; ctDNA, circulating tumor DNA; HRRm, homologous recombination repair mutation; RECIST, Response Evaluation Criteria in Solid Tumors; PCWG3, Prostate Cancer Working Group 3.

## Data Availability

No new data were created or analyzed in this study. Data sharing is not applicable to this article.

## Abbreviations

The following abbreviations are used in this manuscript:

ADT	Androgen deprivation therapy
AR	Androgen receptor
ARPI	Androgen receptor pathway inhibitor
CRPC	Castration-resistant prostate cancer
DDR	DNA damage response
DSB	Double-strand break
HR	Homologous recombination
HRR	Homologous recombination repair
HRRm	Homologous recombination repair mutation
mCRPC	Metastatic castration-resistant prostate cancer
mCSPC	Metastatic castration-sensitive prostate cancer
MRD	minimal residual disease
NHEJ	Non-homologous end joining
ORR	Objective response rate
OS	Overall survival
PARP	Poly(ADP-ribose) polymerase
rPFS	Radiographic progression-free survival
SSB	Single-strand break
